# Using realist evaluation to open the black box of knowledge translation: a state-of-the-art review

**DOI:** 10.1186/s13012-014-0115-y

**Published:** 2014-09-05

**Authors:** Katherine L Salter, Anita Kothari

**Affiliations:** Graduate Program, Health and Rehabilitation Sciences, Elborn College, Western University, London, N6A 1H1 ON Canada; School of Health Studies, Western University, London, ON Canada; Schulich Interfaculty Program in Public Health, Western University, London, ON Canada

**Keywords:** Realist evaluation, Knowledge translation, State-of-the-art review

## Abstract

**Background:**

In knowledge translation, complex interventions may be implemented in the attempt to improve uptake of research-based knowledge in practice. Traditional evaluation efforts that focus on aggregate effectiveness represent an oversimplification of both the environment and the interventions themselves. However, theory-based approaches to evaluation, such as realist evaluation (RE), may be better-suited to examination of complex knowledge translation interventions with a view to understanding what works, for whom, and under what conditions. It is the aim of the present state-of-the-art review to examine current literature with regard to the use of RE in the assessment of knowledge translation interventions implemented within healthcare environments.

**Methods:**

Multiple online databases were searched from 1997 through June 2013. Primary studies examining the application or implementation of knowledge translation interventions within healthcare settings and using RE were selected for inclusion. Varying applications of RE across studies were examined in terms of a) reporting of core elements of RE, and b) potential feasibility of this evaluation method.

**Results:**

A total of 14 studies (6 study protocols), published between 2007 and 2013, were identified for inclusion. Projects were initiated in a variety of healthcare settings and represented a range of interventions. While a majority of authors mentioned context (C), mechanism (M) and outcome (O), a minority reported the development of C-M-O configurations or testable hypotheses based on these configurations. Four completed studies reported results that included refinement of proposed C-M-O configurations and offered explanations within the RE framework. In the few studies offering insight regarding challenges associated with the use of RE, difficulties were expressed regarding the definition of both mechanisms and contextual factors. Overall, RE was perceived as time-consuming and resource intensive.

**Conclusions:**

The use of RE in knowledge translation is relatively new; however, theory-building approaches to the examination of complex interventions in this area may be increasing as researchers attempt to identify what works, for whom and under what circumstances. Completion of the RE cycle may be challenging, particularly in the development of C-M-O configurations; however, as researchers approach challenges and explore innovations in its application, rich and detailed accounts may improve feasibility.

**Electronic supplementary material:**

The online version of this article (doi:10.1186/s13012-014-0115-y) contains supplementary material, which is available to authorized users.

## Background

Knowledge Translation, according to the Canadian Institutes of Health Research, may be defined as the `exchange, synthesis and ethically-sound application of knowledge within a complex system of interactions among researchers and users - to accelerate the capture of the benefits of research for Canadians through improved health, more effective services and products, and a strengthened healthcare system' [1]. As such, it is concerned with more than simple dissemination of research-based information and moves toward application of knowledge in practice [2],[3]. However, facilitating a complex system of interactions within complex healthcare environments is likely to require strategic and equally complex intervention. Complex interventions, like those undertaken for the purpose of knowledge translation, introduce resources, such as knowledge, information or opportunity, and depend upon human reaction and reasoning on the part of both the intervention recipients and the individual(s) providing the intervention to achieve outcomes [4]-[6]. Under these conditions, traditional evaluation efforts that focus on aggregate effectiveness represent a vast oversimplification of both the environment and the interventions themselves [7].

Traditional evaluation efforts attempt to provide an estimate of program effectiveness through the assessment of one or more outcomes, often established *a priori*[4],[8],[9]. Scriven referred to this type of evaluation as `black box' [10]; focused on outcomes, evaluative conclusions are made with no explanation or understanding required with regard to how recorded outcomes might have been produced. The selection and application of black box or aggregate evaluation may have been influenced by experimental models used to examine efficacy of interventions under controlled conditions [11]. In these settings, a relatively small number of carefully selected outcomes are assessed based on the anticipated effects of a limited number of variables controlling for the effects of identified confounders [11]. However, in the real world, this over-simplified model of assessment provides little information about the effectiveness of complex interventions within uncontrolled, context-rich settings and may be insufficient to inform future implementation efforts [4],[5],[7],[12].

Theory-based or theory-driven approaches provide an alternative to black box evaluation that examine not only outcome, but also the possible causes and contextual factors associated with change [13]. Theory-driven evaluation may be defined as any approach or strategy that integrates the use of theory in the conceptualization, design, conduct, interpretation and application of evaluation [14]. Ideally, it should not only generate insight with regard to program effectiveness, but also explain possible underlying causal mechanisms based on postulated associations between program inputs, mediating factors and program outputs [14]. Since theory-driven evaluation, of which realist evaluation is a specific example, is intended to reveal the inner mechanisms by which a program operates, it has been referred to as `white box' evaluation [10],[15],[16].

### Realist evaluation

Although theory-driven evaluation may not be associated with any particular ideology or philosophy [14], in their seminal work, Pawson and Tilley describe realist evaluation (RE) as an explanation-driven, generic approach to evaluation grounded in scientific realism [15]. Scientific realists assume that reality exists separate from and independent of one's perception of it [6],[15],[17],[18]. The goal of scientific realism is to examine regular patterns that exist within reality and offer a more comprehensive understanding of these patterns by providing in-depth explanations through the exploration of generative causal mechanisms, which are sensitive to contextual and social influences [13],[16]-[18]. It is recognized that perfect understanding of reality is not possible; however, as knowledge is emergent, over time one might contribute to what is understood [17],[18].

RE is not a method or a technical procedure; rather it is a logic of inquiry that attempts to answer the question, `What works, for whom, in what circumstances…and why?' [9],[15],[19]. This is accomplished through the identification and examination of underlying generative mechanisms (M) associated with the intervention or program, the conditions or contexts (C) under which the mechanisms operate, and the pattern of outcomes (O) produced [4],[9],[17]. Program or intervention mechanisms are not viewed as equivalent to program components; rather, they are an attempt to represent how program resources are received, interpreted and acted upon by the participant to produce an outcome or pattern of outcomes [6],[9],[15]. However, it is a standard realist proposition that the relationship between generative mechanisms and their effects is not fixed, but is contingent on contextual conditioning [15]. This, Pawson and Tilley suggest, may be expressed as linked C-M-O configurations (or C + M = O) [15].

**Figure 1:**
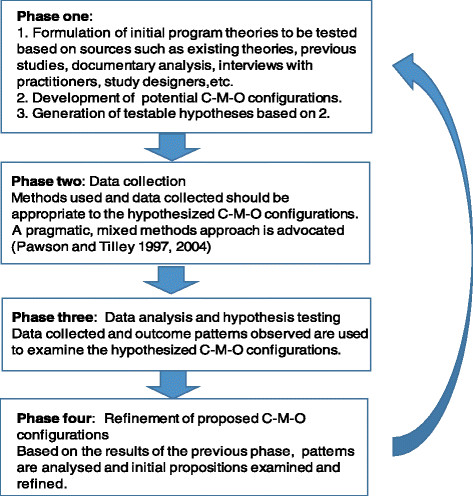
**Phases of the realist evaluation cycle.**

While there is neither a standardized formula, nor a series of requisite steps for producing a realist evaluation, there are hallmark characteristics associated with a realist evaluation. Specifically, the evaluation should a) have an explanatory focus, b) investigate linked configurations of context(s), mechanism(s) and outcome(s), and c) use multiple, mixed methods of data collection to do so [19]. Pawson and Tilley suggest that the process of RE itself proceeds according to a traditional cycle of hypothesis generation, testing and refinement (see Figure 1) [9],[15].

It is not expected that the end result of a realist evaluation will represent a complete explanation of all possible patterns of outcomes associated with the program or intervention studied, or even that the refined C-M-O configurations will provide generalizable representations of what works, for whom and in what circumstances [15]. Instead, it is suggested that RE operates at a middle range, using concepts and data that lie between the description and hypotheses of day-to-day implementation and a universal `theory' [9]. It is anticipated that the mid-range theories produced through the process of program specification or C-M-O refinement may contribute to further cycles of inquiry and, therefore, to ongoing theoretical development [15].

### Realist evaluation and knowledge translation

Recent reviews of interventions seeking to improve the uptake or application of research-based knowledge in healthcare practice have included primarily traditional indicators associated with aggregate assessment of program effectiveness, including observable practice changes such as number of tests or examinations ordered, or other similar, discrete, clinical activities [20],[21]. However, conventional aggregate assessment of intervention effectiveness offers little insight with regard to potential generative mechanisms within a complex learning environment.

A recent review examined the use of RE in health systems research and reported that the technique was slowly `gaining traction' in a broad variety of application contexts [13]. It is the aim of the present review to describe the current state of the literature with regard to the use of realist evaluation within one such context, that is, the application of knowledge translation strategies in healthcare. Application of RE within this context may help to advance understanding with regard to how knowledge translation interventions operate, and provide opportunity for testing and development of theory in this area.

This review will focus on three core elements of RE as identified by Pawson and Manzano-Santaella [19], in addition to challenges reported in the application of the RE framework as a means to address the feasibility of the approach in the assessment of knowledge translation strategies implemented within complex healthcare environments by addressing the following questions: a) Are realist evaluations being conducted to examine knowledge translation interventions in healthcare? b) If so, do authors report the development of C-M-O configurations? c) Do they use mixed methods to investigate hypotheses derived from conjectured C-M-O configurations? d) Do they attempt to explain what worked for whom and under what circumstances, and e) What challenges were experienced by researchers in conducting realist evaluations, and how were these challenges met?

## Method

To examine the application of RE in the study of knowledge translation interventions, a state-of-the-art-review was conducted. A state-of-the-art review is designed to address matters of current interest and activity [22]. As such, it may be considered a form of rapid scoping review, described by Arksey and O'Malley, performed to `examine the extent, range and nature of research activity'[23]. Given that it may be classified as a form of scoping review, we have based our method on the 5-stage scoping study framework proposed by Arksey and O'Malley, within which we will a) identify research questions (above), b) identify relevant studies, c) select studies, d) chart data, and e) collate, summarize and report results [23]. As this was a state-of-the art review, intended to provide a snapshot of the current literature only, and not a full scoping review, it was decided not to perform the additional consultation exercises discussed by Arksey and O'Malley.

To identify articles for inclusion, multiple electronic databases (Medline, SCOPUS, CINAHL and EmBASE) were searched from 1997 (the date of the seminal publication by Pawson and Tilley) to June 2013 using the following terms (`realist evaluation' OR `realistic evaluation' OR `theory-driven evaluation' OR `theory-based evaluation') AND (`knowledge translation' OR `knowledge transfer' OR `knowledge exchange' OR `knowledge management') AND (`health' OR `healthcare') as keyword search strategies. Databases were selected to achieve broad coverage in healthcare without significant duplication. For example, a preliminary search of PsycINFO revealed no articles that were not already retrieved through other sources. Search strategies using only MeSH subject headings were attempted, but returned extremely broad results. Decisions with regard to selection of databases and keyword search strategies employed were made in consultation with a research librarian. Searches were limited to English language publications with human participants. Review articles examining knowledge translation strategies in healthcare were retrieved and their reference lists hand searched, as were the reference lists of all primary studies identified for inclusion. Citations reported in the review of RE application in health system research conducted by Marchal and colleagues [13] were examined for possible inclusion in the present review. Further, issues of `Implementation Science' were searched for the period beginning January 2007 and ending June 2013.

### Study inclusion

Primary studies examining a) the application or implementation of knowledge translation interventions or strategies b) using realist evaluation c) within healthcare settings were identified for inclusion, first by review of title and abstract, then by review of full article texts. Definitions of KT, KT application studies, and interventions provided by Straus and colleagues [2], McKibbon and colleagues [24],[25] and the collective expertise of http://whatiskt.wikispaces.com[26] were used to develop criteria whereby studies could be identified as describing a knowledge translation intervention or strategy. For the purposes of the present review, to be considered a KT study, the source document must describe an intervention or action component representing the application or implementation of knowledge previously unused in the practice setting. Descriptions of both `application' and/or `implementation' were considered for inclusion, as authors may assign various meanings to each of these labels, and implementation may imply deeper commitment to integration in the practice setting (*e.g*., attention to context and other barriers). Further, the intervention must include more than simple knowledge dissemination, which may be considered insufficient to ensure application of knowledge in practice [2]. In addition, the intervention should describe a method that would promote the movement of a specific body of knowledge or research evidence into practice within a specific setting [24]. Examples of intervention terminology included, but were not limited to, guideline implementation, audit and feedback, communities of practice, and knowledge brokers [25],[26]. Health services research that did not describe an intervention or strategy as described above, but did describe changes to the healthcare system (*e.g*., reorganization or redistribution of service delivery or restructuring of human resources) were excluded from the present review.

### Review/summary process

To address the identified review questions, information was abstracted from each study identified for inclusion to address the core elements of the RE framework as follows: a) identification of linked C-M-O configurations to inform testable hypotheses, b) the use of multiple and/or mixed methods to interrogate the proposed C-M-O configurations, and c) explanatory focus; that is, did the study attempt to explain outcomes in terms of underlying mechanisms and contextual influences to present findings that helped to explain how the intervention might or might not have worked, for whom and under what circumstances [19]. In order to address feasibility of RE, all information reported with regard to challenges associated with its application [4],[13] and the ways in which authors have attempted to address the challenges they encountered was also abstracted from each source document.

## Results

**Figure 2:**
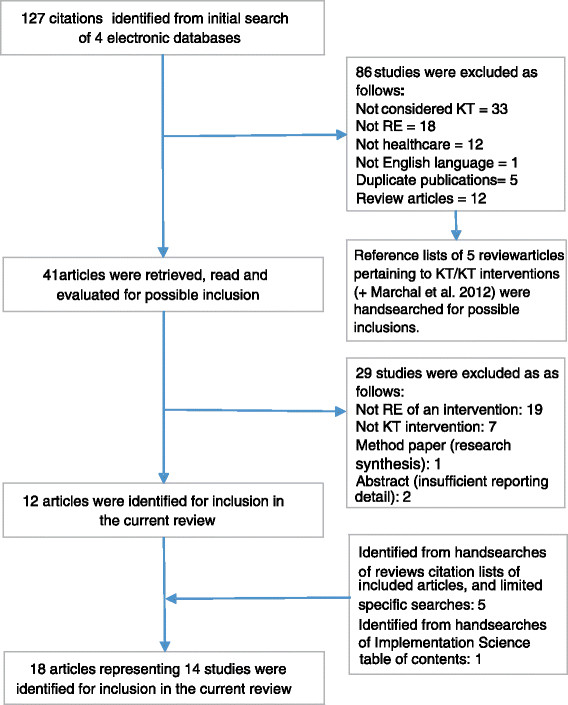
**Results of literature search.**

From 127 citations retrieved from searches performed in 4 databases, a total of 18 articles representing 14 studies were identified for inclusion. Details of the selection process are provided in Figure 2. In two cases [27],[28], the article identified in the initial search provided a description of a realist evaluation associated with a randomized controlled trial. Additional information about these projects and application of the RE approach was sought by identifying additional publications pertaining to these trials [29]-[31]. In a single case, the initial search identified the final step of a realist evaluation [32]. An earlier publication associated with the same project, and providing additional information, was also retrieved [6].

The number and type of studies identified suggest that the application of realist evaluation in the area of knowledge translation is a relatively recent development. Articles retrieved for inclusion were published in the years between 2007 and 2013. Of the 14 studies identified, 6 were protocols that contained descriptions of proposed rather than completed research [28],[33]-[37]. In general, there did not appear to be any specific preference in terms of healthcare setting or type of knowledge translation intervention studied. Projects were conducted in a variety of settings and examined an assortment of interventions including a) provision of information, b) training and use of facilitators or intermediaries, c) knowledge brokers, d) guideline or protocol implementation strategies including the use of care pathways, e) communities of practice, and f) implementation of evidence-based interventions to improve care. Additional details of articles retrieved for inclusion are provided in Table 1.

**Table 1:** **Articles selected for inclusion**

Authors, year, country	Country of origin	Study type	Setting	KT intervention
Goicolea *et al.*, 2013 [33]	Spain	Protocol	Primary healthcare teams	Provision of guidelines, protocols, training and information systems
Chouinard *et al*. 2013 [34]	Canada	Protocol (RCT)	Primary care	Implementation of an evidence-based intervention to improve chronic-disease management
Seers *et al*. 2012 [35]	Europe	Protocol (RCT)	Long-term nursing care	Facilitation interventions vs. standard dissemination of information in evidence-based practice
Ranmuthugala *et al*. 2011 [36]	Australia	Protocol	Healthcare settings	Communities of practice
				(*Note: Communities of practice will be studied using an opportunistic sample*)
Rycroft-Malone *et al*. 2011 [37]	UK	Protocol	Various healthcare settings	Communities of practice/implementation teams
				*(Note: This study is part of work undertaken within the Collaborations for Leadership in Applied Health Research and Care [CLAHRC]. Specific settings are to be determined.)*
Mackenzie *et al*. 2009 [28],[30],[31]	UK	Protocol (RCT)/Evaluation	Local health or community centres	Implementation of an evidence-based intervention to improve smoking cessation by improving weight management through nutritional/lifestyle intervention
Williams *et al*. 2013 [38]	UK	Evaluation	Hospital	Intermediary program (where intermediary was defined as `*a linking agent, facilitator, change agent, champion or opinion leader*')
Ward *et al*. 2012 [39]	UK	Evaluation	Large mental health organization	Knowledge brokering within service delivery teams
Moore *et al*. 2012 [27],[29]	UK	Evaluation (RCT)	Primary healthcare	Implementation of a policy-mandated element of a national guideline
Wand *et al*. 2011 [6],[32]	Australia	Evaluation	ED-based outpatient service	Implementation of an evidence-based intervention to improve access and acceptability of mental health care
Rycroft-Malone *et al*. 2010 [40]	UK	Evaluation	Multiple clinical sites	Implementation of protocol-based care (guidelines, care pathways, algorithms, protocols)
Bick *et al*. 2009 [41]	UK	Evaluation	Birth Centre	Implementation of care pathway
Wiechula *et al*. 2009 [42]	Australia	Evaluation	Acute care hospital	Implementation of Knowledge Translation Toolkit
Tolson *et al*. 2007 [43]	UK	Evaluation	Rural primary care	Implementation of guidelines within a managed care network

### The development of C-M-O configurations

Nine of the included studies made reference to C-M-O configurations in reporting the application of realist evaluations [28],[32],[33],[36]-[38],[40],[41],[43]. Four of these were study protocols that provided detailed descriptions of initial investigations that included searches of existing literature, interviews and document analysis intended to inform identification of contextual factors, possible underlying mechanisms and outcome patterns that could be used to develop C-M-O configurations from which to generate study hypotheses [28],[33],[36],[37]. The report provided by Mackenzie and colleagues [28] presented the results of this first stage of evaluation and also provided examples of proposed C-M-O configurations. For example, `participants who are motivated and share a good rapport' (context) may `share ideas, experiences and support one another' (mechanism), which may be associated with a smoking cessation and improved weight management (outcome) [28].

Of the remaining five studies, the development of C-M-O configurations was noted as part of the initial stages of realist evaluation in four [32],[40],[41],[43]. A single study provided contextual factors, mechanisms and outcomes produced within an integrated conceptual framework and then provided conjectured C-M-O configurations as a study finding [38]. Given that there are no standardized, methodological guidelines available for the application of RE [19],[44], it is not surprising that the ways in which C-M-O configurations were developed and applied varied within the relatively few completed evaluation studies that reported their use.

### Context

Pawson and Tilley define context as `features of the conditions in which programs are introduced that are relevant to the operation of program mechanisms' [9]. One is cautioned to avoid equating context with location or setting; instead, one is encouraged to examine roles and relationships, technology, economic conditions, demographics, and so on [9]. Two studies provided definitions of context taken directly from the works of Pawson and Tilley [38],[41], while a third [32] relied upon the following definition `the background circumstance that encourages or enables a particular group of stakeholders to be assembled for negotiation' [6]. Rycroft-Malone and colleagues referred to context as the different clinical settings in which the program, in this case protocol-base care, was used [40]. However, these authors also adopted the use of a specific, knowledge translation framework, PARiHS (the Promoting Action on Research Implementation in Health Service framework), which is intended to facilitate the identification of contextual factors that may influence knowledge uptake [45]. Specifically, the PARiHS framework identifies contextual sub-elements as culture, leadership and evaluation. Use of this framework may have influenced their conceptualization of the implementation contexts and the contextual factors, under investigation.

### Mechanism

Program mechanisms may be described as the underlying processes that describe how an intervention or program produces change [4]. Mechanisms explain the impact of the program of intervention resource(s) on the individual's reasoning and their choices [9],[15]. Four of the five studies identified as noting the development or use of C-M-O configurations also relied on definitions of mechanism based on this one, provided originally by Pawson and Tilley, to guide their process [32],[38],[40],[43]. Bick and colleagues [41] equated mechanisms with the introduction of `appropriate ideas and opportunities', which was also proposed by Pawson and Tilley [15].

### Outcome

Any program is likely to result in a mixed pattern of outcome consisting of both intended and unintended consequences [9]. Outcomes may take many forms, and programs or interventions should be tested against a range of carefully conceived indicators of these potential outcomes, including before and after assessments of change where possible [9],[19]. All five evaluation articles that provide C-M-O configurations include outcomes; however, none of these are well-defined or accompanied by indications of how they might be assessed. In a single case, Mackenzie and colleagues provided examples of proposed C-M-O configurations derived from the initial stages of their project [28]. In the table provided, the outcome associated with each statement was represented as simply positive (success) or negative (failure) defined in terms of weight management and smoking cessation. The information pertaining to the assessment of these variables is available in the publications pertaining to the associated randomized study [28],[30]. It should be noted, however, there were no conjectured outcomes that represented potential changes in knowledge translation outcomes of interest related to conceptual knowledge, such as changes in level of knowledge, understanding or attitudes that could be reasonably associated with potential generative mechanisms within a knowledge-focused intervention [46].

### Initial CMO configurations

Of the five studies providing initial C-M-O configurations, two provided statements only, in which the separate C, M and O components were not identified explicitly. Rycroft-Malone and colleagues provided multiple propositions in 4 identified areas of theory, but did not specify each element of the proposition statements [40]. Bick and colleagues offered a single all-encompassing statement of C-M-O in which the program was presented as the mechanism, the setting as the context, and the overall impact of the program as the outcome [41]. Although there is certainly no hard and fast rule regarding the reporting format for C-M-O configurations, given reported difficulties in defining and identifying mechanisms and in differentiating mechanisms from context [13], the use of the C + M = O table suggested by Pawson and Tilley [9],[15] might have served to provide clarity in complex evaluation environments with many contextual factors, mechanisms, and patterns of outcome possible. Three studies did provide graphic and/or tabular depictions of C-M-O configurations [6],[32],[38],[43]. However, in those studies, linked C-M-O configurations were not provided. Instead, there were lists presented of possible contextual factors, mechanisms and outcomes rather than linked configurations upon which to build testable hypotheses.

### The use of multiple and/or mixed methods

Pawson and Tilley noted that one should be pragmatic in the selection of data collection methods and analyses [15]. While advocating a pluralist and pragmatic approach to the selection of methods, encompassing strategies associated with the collection of both qualitative and quantitative data, Pawson and Tilley also noted that the choice made should be appropriate to the hypotheses generated [9],[15],[19]. Pawson and Manzano-Santaella suggest a balanced approach, noting that the investigation of each component in the C-M-O configuration might favour input from different data sources [19]. Context, for instance, might be investigated using strategies that focus on the collection of comparative or historical data while mechanisms might be illuminated via qualitative data and outcomes assessed quantitatively [19].

All of the studies identified for inclusion in the current review reported the use or intended use of multiple methods of data collection (see Table 2). The most commonly reported forms of data collection were strategies associated with the collection of qualitative data such as interviews, document review, and observation techniques. In addition, strategies to collect quantitative data were reported by nearly one-half of the studies, apart from whatever might have be included in the routinely collected local data, clinical records, or patient chart audits. In several of these cases, quantitative assessment was conducted or proposed as part of the randomized controlled trial with which the realist evaluation was associated [28],[34],[35]. In two of these reported protocols, the degree to which the quantitative assessment might be integrated with the realist evaluation was not clear from the information presented, and no conjectured C-M-O configurations were available for either project when the protocols were published [34],[35]. In the case of Mackenzie and colleagues, the outcomes referred to in the C-M-O configurations were quantitative assessments of weight management and smoking cessation [28]. Then, qualitative data were used to query *a priori* identified mechanisms in an attempt to explain success or failure in terms of weight management and smoking cessation [30]. Similarly, Moore and colleagues assessed change in scores on the Behaviour Change Counselling Index over time and used a process of inductive thematic analysis, taking contextual influences into consideration, to develop theories about why quantitatively assessed changes may or may not have occurred [27].

**Table 2:** **Methods of data collection reported**

Data collection method	Reported in:
Interviews (semi-structured, conversational, open)	Goicolea *et al*. 2013 [33], Chouinard *et al*. 2013 [34], Ward *et al*. 2012 [39], Seers *et al*. 2012 [35], Williams *et al*. 2013 [38], Moore *et al*. 2012 [27], Ranmuthugala *et al*. 2011 [36], Rycroft-Malone *et al*. 2011 [37], Wand *et al*. 2011 [32], Rycroft-Malone *et al*. 2010 [40], Bick *et al*. 2009 [41], Mackenzie *et al*. 2009 [28], Wiechula *et al*. 2009 [42], Tolson *et al*. 2007 [43]
Focus groups	Goicolea *et al*. 2013 [33], Chouinard *et al*. 2013 [34], Tolson *et al*. 2007 (executive review sessions) [43]
Document review	Goicolea *et al*. 2013 [33], Chouinard *et al*. 2013 [34], Ward *et al*. 2012 [39], Seers *et al*. 2012 [35], Williams *et al*. 2013 [38], Rycroft-Malone *et al*. 2011 [37], Rycroft-Malone *et al*. 2010 [40], Bick *et al*. 2009 [41], Wiechula *et al*. 2009 [42], Tolson *et al*. 2007 [43]
Participant or non-participant observation	Goicolea *et al*. 2013, Seers *et al*. 2012 [35], Williams *et al*. 2013 [38], Rycroft-Malone *et al*. 2011 [37], Rycroft-Malone *et al*. 2010 [40], Bick *et al*. 2009 [41]
Field notes, journals, notebooks (observer generated)	Ward *et al*. 2012, Rycroft-Malone *et al*. 2010, Bick *et al*. 2009, Wiechula *et al*. 2009 [42], Tolson *et al*. 2007 [43]
Routinely collected local data, clinical records, audits	Goicolea *et al*. 2013 [33], Seers *et al*. 2012 [35], Rycroft-Malone *et al*. 2011 [37], Wand *et al*. 2011 [32], Wiechula *et al*. 2009 [42]
Quantitative measurement: assessment of defined construct or indicator	Goicolea *et al*. 2013 (presence vs. absence of behaviours, procedures, materials or information) [33], Chouinard *et al*. 2013 (implementation fidelity) [34], Ward *et al*. 2012 (time contributed) [39], Seers *et al*. 2012 (RCT primary study outcome = compliance with recommendations) [35], Moore *et al.* 2012 (ratings of interview technique fidelity before and after training using the Behaviour Change Counselling Index) [27], Wand *et al*. 2011 (self-report measures of psychological distress, self-efficacy and client satisfaction) [32]; Mackenzie *et al*. 2009 (quantitative outcome assessed as part of RCT - weight management and smoking cessation) [28]
Other	Alberta Context Tool - Seers *et al*. 2012 [35]
	Recordings of consultations - Moore *et al*. 2012 [27]
	Surveys and social network analysis - Ranmuthugala *et al*. 2011 [36]
	Discussions with stakeholders - Rycroft-Malone *et al*. 2011 [37]
	Workshop-related data (multimedia recordings, images, documents) - Rycroft-Malone *et al*. 2011 [37]
	Tracking `patient journeys' (contacting patients several times as they moved through the clinical service) - Rycroft-Malone *et al*. 2010 [40]

### Explanatory focus

All of the studies reported justifications for the selection of realist evaluation that cited understanding how and why the program or intervention at hand (the theory incarnate) did or did not work. Realist evaluation provides a model of generative causation in which outcomes may be explained by the action of particular underlying mechanisms in specific contexts; hence, C + M = O [15]. Six of the reports identified for inclusion were study protocols, in which C-M-O configurations had yet to be refined and explanations attempted. Of the five completed evaluation studies reporting conjectured C-M-O configurations or lists at the outset, four presented results that included refinement of proposed configurations and explanations within the realist framework [32],[38],[40],[43]. As was the case for the conjectured C-M-O configurations, the way in which the inductively-derived theoretical explanations were presented varied. For example, Rycroft-Malone and colleagues presented a table containing comprehensive lists of statements separated into categories of a) what worked b) for whom, c) how and, d) in what circumstances that represented considerable revision to the project's conjectured C-M-O configurations [40]. Wand and colleagues used the data collected and the proposed C-M-O configurations to formulate statements of mid-range theory in four domains (*e.g*., `Individuals valued the Emergency Department location, prompt access and flexible appointment times for patient follow-up') [32]. Similarly, Williams and colleagues provided a list of `conjectured' C-M-O configurations, and as in the preceding two examples, did not specify context, mechanism or outcome (*e.g*., `In clinical areas, high levels of intermediary presence and increased attention to intermediary presence leads to modification of behavior') [38]. The study conducted by Tolson and colleagues represented a unique application in that there were three evaluation points. At each round, the conjectured C-M-O configurations were revised by a committee using data (outlining the actual C-M-O process), Nominal Group Technique discussion and reflection, and then were applied again [43].

The report provided by Moore and colleagues did not provide conjectured C-M-O configurations, or refined configurations after evaluation of the intervention, but the authors did describe and report a realist evaluation process whereby study outcomes, assessed quantitatively over time, were examined using qualitative data to develop explanations for why change was (or was not) documented [27]. The results were presented in a matrix of subthemes and change in practice that served to illuminate outcome patterns [27].

### Methodological challenges associated with realist evaluation

It has been suggested that theory-based evaluation intended to investigate and explain mechanisms is both labour- and resource-intensive [17]. Pawson and Tilley noted that RE is an intellectually challenging process [9]. There are no simple steps or strict methodological rules to follow and no standardized approach to take [9],[44]. Challenges or potential challenges associated with undertaking RE were noted in several of the publications included in the present review. Reported challenges are summarized in Table 3.

**Table 3:** **Challenges and limitations reported in the implementation of realist evaluation**

Thematic challenges	Details of challenges reported
Time and resource intensive	• Data collection/demands on participant time and resources (feasibility issue) - revised collection so that survey administration, interviews and discussion demands have been distributed to alleviate demand [36].
	• Identification of an outcome that would demonstrate impact of a COP was difficult [36].
	• Resource intensive. Only one cycle of data collection was possible; more than one would have allowed more refinement of CMO configurations and possible resolution of difficulties defining mechanisms [40].
	• Refinement required flexibility and continual, iterative, process of checking back and forth between configuration and data. Should allow sufficient (ample) time for a process of discussion and debate [40].
	• Flexibility must be required to adapt to the needs of the teams within the specific contexts [42].
	• Difficult to conduct this type of research within available resources [42].
	• Resource and time constraints place limitations on the length of time within which the intervention may run, evaluations must be made and analyses achieved; the authors attempted to include three distinct evaluation points and an ongoing cycle of CMO evaluation and refinement [43].
	• Uncertainty with regard as to the best time to begin evaluation and refinement of the conjectured CMOs; influenced by the nature of the intervention but also by the available resource associated with the project [43].
Lack of previous/existing information or evidence to inform the development of C-M-O configurations	• Existing evidence to inform the development of conjectured CMO was scarce (particularly in team environments). Previous programs did not attempt to make underlying theory explicit [33].
	• Identification of an outcome - difficult given lack of existing evidence [36].
	• Development of initial configurations limited by the amount and quality of available evidence [40].
	• Difficult to define `mechanism' and sometimes to distinguish mechanisms from contextual factors. In addition, simultaneously functioning mechanisms difficult to interpret; defining CMOs as clearly as possible as early in the process as possible might help make refinement easier [40].
Defining contextual factors and/or mechanisms	• Difficulty in identifying potential mechanisms and outcomes; required lengthy discussions and many iterations [36].
	• No clear steps to guide process; operationalization challenging, requiring trial and error [40].
	• Other teams might not produce same refinements; a clear and transparent audit trail was produced, so that others may understand the findings [40].
Defining and assessing outcomes	• Increased, and more complex demands for assessment; must design a means to measure effect sensitive to team culture and values as well as service delivery (feasibility issue). Will use existing data collection where possible [33].
	• Results cannot be used to predict outcomes in the future [42].
	• Difficult to identify adequate indicators of program effectiveness; may only be able to address how the program worked, not if it worked in an effective manner [43].

It was anticipated that its application would result in a substantial number of reported difficulties or limitations. However, relatively few articles (n = 5) included a discussion of study challenges or limitations specific to the application of realistic evaluation. Of the five articles that did report challenges, three offered descriptions of completed studies [40],[42],[43], while the remaining two were study protocols [33],[36]. Most of the challenges expressed were shared by more than the authors of a single study and were related to all phases of the RE cycle. Lack of previous or existing evidence to inform the development of conjectured C-M-O configurations was cited as problematic [33],[40]. There were difficulties expressed in defining both mechanisms and contextual factors and, sometimes, in distinguishing one from the other [36],[40]. The area of outcome and outcome assessment appeared particularly problematic. Authors noted that effectiveness outcomes were difficult to identify and define [36],[43]. In addition, it was perceived that the assessment burden, *i.e*., the workload generated from a realistic evaluation, was complex and increased the burden on available resources as well as on participants [33],[36],[43].

Overall, RE might be considered time consuming and resource intensive. Several authors noted a substantial investment of time required for discussion (particularly during development of initial C-M-O configuration and refinements to proposed configurations), while others reported making adaptations to the RE process in order to work within available project time and resources [36],[40],[42],[43].

## Discussion

Overall, there was a small number of relatively recent reports identified that described either completed or proposed realist evaluations of knowledge translation interventions in healthcare settings. The oldest article identified was published in 2007, a full decade after the seminal work of Pawson and Tilley. The most recent articles identified were study protocols including a large scale, multi-national, pragmatic randomized controlled trial [35] of significantly greater scope than earlier projects reported. These results suggest that, while the body of work in realist evaluation within the knowledge translation literature may be small, it has been growing and will continue to do so. Although the majority of the work to date has been completed by researchers in the UK, there is a small number of reports originating in a limited number of other countries.

The reasons that there were no identified published reports describing the use of RE in the evaluation of knowledge translation interventions in the years prior to 2007 are a matter of speculation. In terms of the study of knowledge translation, the gradual adoption of theory-driven or theory-based approaches to evaluation may simply reflect changes over time in the way in which KT is conceived and investigated. Early reviews of knowledge translation interventions focused on aggregate assessments of effectiveness, often defined as `improvements in care' or `performance' [47]. Increasingly, it is acknowledged that KT is a complex process, occurring within a complex system, for which simple aggregate assessments such as these may be inadequate [7],[48],[49]. In addition, it has been suggested that, in the development of KT interventions, insufficient attention may be paid to either existing theory or contextual factors [50]. The need for ongoing development of theory to understand and continue to improve the development and implementation of specific KT interventions as well as the science of KT, in general, is an important issue that remains the subject of ongoing debate [50]-[52]. Theory-driven evaluation to investigate KT processes represents an inductive method of theory testing and development [50]. In RE, the program or intervention itself is regarded as the theory `incarnate', which is introduced into an existing social context [9],[15]. Pawson stated that evaluation begins by `eliciting and formalising' the theory or theories to be tested by drawing on a variety of information sources that may include `documents, program architects, practitioners, previous evaluation studies and social science literature' [9]. Theory accumulates through an iterative process in which theoretical propositions are constructed and tested using empirical data to investigate patterns of outcomes under specific contextual influences [15],[50].

### Feasibility of realist evaluation in knowledge translation

Although appealing for its theory-building and explanatory focus, adoption may have been limited by challenges associated with the application of realist evaluation. Any form of evaluative research that is intended to unearth underlying program mechanisms is likely to be labour- and resource-intensive and, while interesting and informative, a full realist evaluation may not always be possible or appropriate [10],[17],[53]. Four studies included in the present review noted that application of realist evaluation presented challenges in terms of time and resource management that had to be overcome or accommodated in order to complete the evaluation [36],[40],[42],[43]. Hewitt and colleagues suggested that a flexible approach to RE is common and identified a tendency to begin the RE cycle with the collection of data, omitting the initial phase that includes articulation of program theory, and development of conjectured C-M-O configurations [4]. Certainly, in the present review, study descriptions provided by authors reflect substantial variations in the application of the realist approach. However, in terms of completion of phase one specifically, (as defined in Figure 1), four of the study protocols included proposed development of C-M-O configurations in order to generate study hypotheses [28],[33],[36],[37], and an additional five completed evaluation studies reported some form of C-M-O configuration statement(s) or included a table or graphic depicting lists of elements [32],[38],[40],[41],[43]. Commonly reported adaptations in response to time or resource limitations were elimination of additional evaluation cycles, shortening of time frames or reduction of assessment or data collection requirements rather than elimination of phases from the proposed evaluation framework [33],[36],[40],[43].

### Identifying mechanisms, contexts and outcomes; the challenge of the C-M-O

In general, authors offered comments regarding challenges at all phases of the evaluation process. Many, however, related to the development of the C-M-O configurations and the identification of specific mechanisms, contextual factors and assessment of outcomes - the components required to create each explanatory proposition. For instance, initial development of C-M-O configurations may be hindered by a lack of existing theory, evidence or information [33],[40]. The demands placed on the researcher in this situation may be for a somewhat creative theorist who is capable of taking imaginative leaps in conjecture based on a reasonable understanding of how things might work and commonsense judgment [5],[9],[19],[54]. Realist evaluation is often aimed at helping policymakers or administrators make choices appropriate to their own setting, thus it would seem reasonable to draw on their perspectives to assist with interpretation. Some protocols [27],[29],[33]-[36] and one study [42] mention doing just this through either formal (*e.g*., advisory committees) or informal meetings.

Although often guided by definitions of mechanism provided by Pawson and Tilley, authors reported experiencing difficulty in identifying underlying mechanisms and distinguishing them from either program components or from contextual factors [33],[36],[40]. Further, Rycroft-Malone and colleagues expressed difficulty in the interpretation of potential, interactive mechanisms that functioned simultaneously [40]. Pawson and Manzano-Santaella point out that each program or intervention may be represented by multiple theories, each of which may have many generative mechanisms functioning simultaneously [19]. In addition, not all mechanisms will be functioning in association with all possible contextual factors [53]. The development of linked, propositional, conjectured C-M-O configurations addresses these very specific pathways through the examination of outcomes generated [9],[15],[53].

Outcomes, like mechanisms and contextual factors, should be carefully conceived and well-described [19]. It has been suggested that researchers not rely on qualitative data alone for the assessment and comparison of hypothesized outcome; instead, it has been recommended that the assessment of a variety of outcomes and employing various measures, including quantitative, might be less likely to produce artificial results [9],[19],[54]. Several authors did note challenges associated with the identification and definition of study outcomes [36],[43] as well as with managing a perceived increase in the burden of assessment associated with realist evaluation [33],[36],[43]. Although the authors reported the use of multiple and mixed methods for data gathering, completed evaluation studies included in the present review provided relatively little information about how the outcomes identified *a priori*, as part of the development of proposed C-M-O configurations, were defined and assessed as part of the hypothesis testing process described by Pawson and Tilley [15]. If an essential component in the explanation-building work of realist evaluation is a process of refinement that seeks to test conjectured propositions, moving back and forth from theory to empirical data [53], then the definition of outcome and the understanding of how to collect data appropriately to inform that outcome is crucial [15],[19].

### Integrating other frameworks

Given the challenges associated with the identification of contextual factors, underlying mechanisms and potential mechanisms in the articulation of program theory, coupled with a relative lack of guidance with regard to how to conduct a realist evaluation, the integration of additional structural tools, such as conceptual frameworks, may serve to facilitate the collection and organization of information researchers need to consider in their analysis of the implementation environment. Conceptual frameworks, in general, may facilitate the identification of important variables and associations between variables that should be included in the development of explanation-building propositions [49]. One such conceptual framework is the PARiHS, a knowledge-implementation-specific framework intended to facilitate the identification of an association between factors that may influence the uptake of knowledge [45]. The developers of the PARiHS framework have suggested that the use of theory-driven evaluative approaches such as realist evaluation together with the PARiHS conceptual framework may serve to elucidate some of the complexities of knowledge translation and avoid the reductionism that could result from reliance on diagnostic, score-based approaches to measurement in KT [49]. While a total of six studies included in the present review cited the integration of other, complementary conceptual frameworks, the only one used repeatedly was the PARiHS framework; cited by a total of four studies [34],[35],[37],[40].

### Limitations

The present review was intended as a snapshot only, to provide a sense of the current state of the art of realist evaluation as applied in the investigation of KT interventions and reflected in the available literature. It was not intended as a comprehensive scoping or systematic review. It is acknowledged that a more extensive search including more databases or grey literature sources and exploring interventions related to KT (*e.g*., evidence-based practices or quality improvement), as well as considering the inclusion of articles published in languages other than English may have identified reports that might have reflected a broader application of realist evaluation in a wider variety of settings. However, the search results obtained were similar in pattern to a previous review in the area of health systems research [13], suggesting that the articles retrieved may be a reasonable representation of the current state of the art. The identification of KT interventions also proved to be difficult. This is not a well-defined terminology, and KT is not easily operationalized. In the end, multiple sources were used to create as inclusive, yet specific, a definition as possible while preserving the central notion of the application or implementation of previously unused knowledge.

The present study examined whether or not researchers reported the inclusion of certain, core RE elements, and was not intended to represent an evaluation of the methodological quality of individual realist evaluations. As such, we did not examine whether the methods used to generate program theory, derive hypotheses, or gather data were appropriate or consistent. There is no standardized framework or structured guidance on how to conduct a realist evaluation and no agreed-upon criteria available by which to judge the quality of a completed study that are specific to RE.

### Considerations for future research

Based on the state-of-the-art snapshot afforded by the present review, the application of realist evaluation to the study of KT interventions beginning with the articulation of mid-range theory and the development of linked C-M-O configurations appears possible. However, the process by which this is done may be difficult, time-consuming, and uncertain [9],[40],[44]. Unfortunately, relatively few authors provided descriptions of challenges experienced or of the solutions they created to meet specific challenges. Several of the studies identified here were written by the same author or group of authors and represent little geographic or cultural variation in setting. If the use of realist evaluation is to become more widespread, more detailed information about its use, and the challenges associated with it, would be of great interest to researchers who intend to pursue it, given that there is no standardized guideline or set of rules to follow, and that both innovation and common sense are highly valued within the RE paradigm [8],[19],[53].

## Conclusion

A small, relatively new body of published works was identified for inclusion in the present review, suggesting that the use of inductive, theory-building approaches to evaluative research, such as realist evaluation, may be slowly taking hold in the area of knowledge translation. The explanatory focus of realist evaluation is closely allied to the development of linked C-M-O configurations. However, the development of conjectured configurations appears difficult, particularly with respect to the identification of potential mechanisms and the definition of outcome(s). Despite challenges, undertaking this evaluation approach is possible given that authors undertook all stages of the evaluation, included the core elements in their work, and completed examples providing rich information, including configurations for further investigation of what type of KT interventions work for whom, in what context, and why. In addition, protocols for future realist evaluations indicate ongoing investment in this theory-driven approach. Continued exploration and innovations in realist evaluation, including integration of other conceptual frameworks in aid of illuminating important aspects of the implementation context, such as the PARiHS network, should include detailed accounts, where possible, of the challenges encountered and solutions created.

## Authors' contributions

KS was responsible for initial development of the paper's concept, conducting the search, identifying studies for inclusion, information abstraction, and compilation of findings. KS wrote the manuscript and was responsible for its subsequent editing and revision. AK participated in developing and refining the concept for this work, assisted in the development of the inclusion criteria, acted as a consultant to determine study inclusion, and provided feedback and editing assistance. Both authors read and approved the final manuscript.

## Authors' information

KS is a PhD candidate in health promotion, whose primary interest lies in knowledge translation. AK is an associate professor in the School of Health Studies with a CIHR new investigator award in knowledge translation.

## Authors’ original submitted files for images

Below are the links to the authors’ original submitted files for images. Authors’ original file for figure 1 Authors’ original file for figure 2
