# Supplementary figures and images for: Using realist evaluation to open the black box of knowledge translation: a state-of-the-art review
Source: Implement Sci. 2014 Sep 5;9:115. doi: 10.1186/s13012-014-0115-y (PMC4172789; doi:10.1186/s13012-014-0115-y)

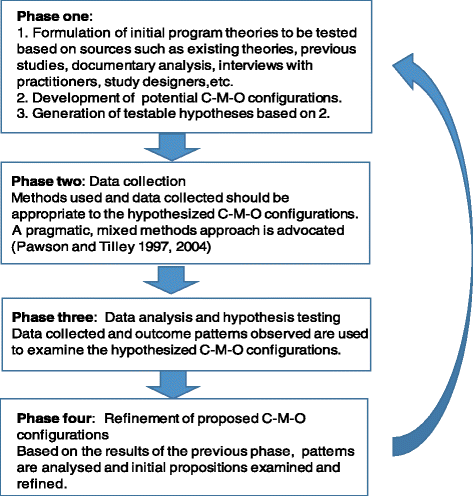

Supplement: Supplementary file 1 — Authors’ original file for figure 1 [file 13012_2014_115_MOESM1_ESM.gif]

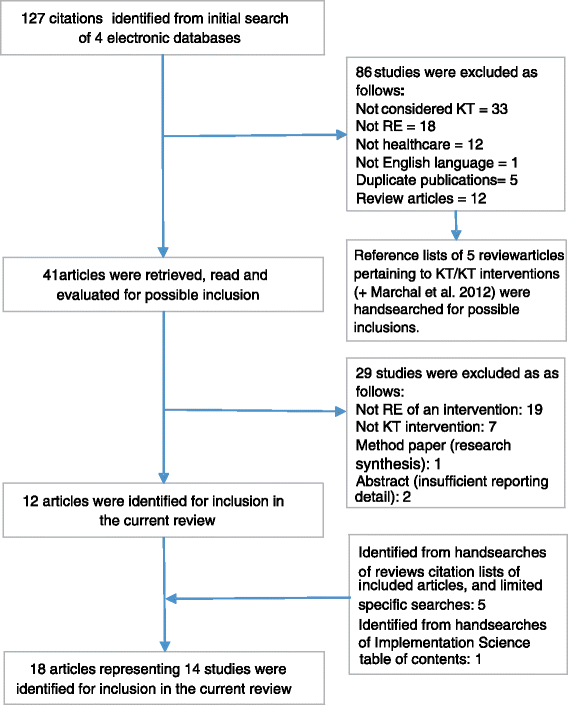

Supplement: Supplementary file 2 — Authors’ original file for figure 2 [file 13012_2014_115_MOESM2_ESM.gif]
